# Tiered Levels of Resting Cortisol in an Athletic Population. A Potential Role for Interpretation in Biopsychosocial Assessment?

**DOI:** 10.3390/jfmk4010008

**Published:** 2019-01-21

**Authors:** Billymo Rist, Alan J. Pearce

**Affiliations:** School of Allied Health, La Trobe University, Melbourne 3083, Australia

**Keywords:** Cortisol, psychological traits, athlete stress

## Abstract

Background: Cortisol is a steroid hormone that follows a distinct diurnal timeline; however, while healthy ranges exist, it not been determined whether or why individuals differ on baseline cortisol levels. The aim of this study was to test the anecdotal evidence of different levels of responders by classifying these levels in resting cortisol, and to correlate individual cortisol responses to psychological traits. Methods: Twenty-two male athletes (mean age 22.5 ± 4.34 years) provided two saliva samples at the same time each day over three days in week one of their pre-season to determine individual baseline salivary cortisol levels. Participants also completed self-report psychological traits measures for correlation to cortisol levels. Results: Three levels of cortisol in responders were clearly identified (*F*_2,19_ = 69.00, *p* < 0.001). Pearson’s correlation coefficient showed that there was no significant relationship between baseline cortisol levels and psychological traits (optimism, *r* = 0.23, *p* = 0.29; stress, *r* = 0.05 *p* = 0.82; decision making, *r* = 0.19 *p* = 0.38). Conclusions: This novel study identified that within an overall healthy range, individual athletes will likely fall into either a low, average or high band of baseline cortisol. However individual responses did not correlate to self-report psychological traits. Caution is required if sports science staff wish to use cortisol to measure psychological stress.

## 1. Introduction

Cortisol is the steroid hormone produced by the adrenal glands. As well as maintaining alertness and sleep-wake cycles, adrenocorticotropic hormone (ACTH) released in the brain triggers the release of cortisol and adrenaline in the adrenal glands, in response to stressful or threat-like experiences. Involved primarily in the stress response, cortisol release counters this threat by providing increased metabolism providing increased energy [1].

Circulating levels of cortisol are usually described as time-based healthy ranges, allowing for the determination of abnormal cortisol levels observed in clinical physiological conditions, such as Addison’s Disease or Cushing Syndrome. Cortisol is also used to measure stress responses relating to physical lifestyle habits, including sleep patterns, nutrition and exercise. However, cortisol release has also been shown in psychological stress, including those suffering with anxiety or depressive disorders [2,3].

Whilst most work has been completed in the wider population, the issue of chronic stress in athletes is an area of emerging study in the sports science [4]. Many professional sporting clubs are now taking chronic stress in elite athletes more seriously by employing sports psychologists, or so-called ‘wellbeing managers’, to provide ongoing support for full-time athletes, who not only must deal with stressors in the field, such as the repetition of constant training [5], and ongoing injuries, but also off the field, such as scrutiny from fans, and social media [6]. For example, Parmigiani et al. [7] demonstrated in a cohort of male Karate athletes, mean age 26.7 ± 7.9 years, that those with higher levels of cortisol had higher levels of risk taking traits and emotional insecurity. Conversely, Lamb et al. [8] compared female collegiate athletes in the off-season with those in-season showing no difference in cortisol levels between off-season athletes who reported higher anxiety, to those in-season.

While cortisol measures investigating the physiological aspects of sport is not a new concept, using cortisol for psychological monitoring in athletes is relatively novel [9]. As a result, whilst many support staff involved in professional sports clubs may be aware of ‘healthy ranges’, they may not be aware of the anecdotal evidence of ‘low’, ‘moderate’, and ‘high’ cortisol responders, all of which are within healthy ranges. Whilst the mechanisms contributing to different responders has not yet been fully explored, those naive to employing cortisol measures to measure psychological stress in athletes could be in jeopardy of over interpreting ‘high’, or alternatively ‘low’, responders having psychophysiological stress.

Therefore, the aim of this study was to test the anecdotal evidence of different levels of responders by classifying these levels in resting cortisol using one professional sporting team. A secondary aim was to correlate individual cortisol responses to psychological traits. We hypothesized that in one professional team where athletes are from the one sport and are undertaking a similar training regime there would be clearly delineated low, moderate, and high cortisol responses. We also hypothesized that individual cortisol responses would significantly correlate to psychological traits measured. 

## 2. Materials and Methods 

### 2.1. Recruitment

As part of a larger study examining psychophysiological correlates in professional Australian football athletes responding to injury and approaches to rehabilitation [10], a convenience sample of 22 professional athletes were invited to participate in the study. Inclusion criteria included professionally contracted Australian Football League (AFL) players aged 18 years or older. Participants were excluded if they were injured. Data was collected during the off-season period prior to the commencement of heavy load physical activity pre-season training. All methods were approved by the University Human Research Ethics Committee (HEC18041), the AFL team gave organisational consent, and participants gave informed consent prior to data collection.

### 2.2. Cortisol Measures

Athletes were instructed to complete two baseline salivary cortisol measures over a three-day period, with each sample collected within 15 min after waking up, and one day between the first and second sample. For the collection of saliva, participants were required to place the oral fluid collector swab in their mouth on top of the tongue and close their mouth. Participants were instructed to continue swabbing until the indicator on the swab has turned blue in color and 0.5 mL of saliva was obtained. Saliva collection took approximately 30 s. 

To measure each participant’s cortisol levels, a portable salivary testing device was used (iPRO lateral flow device, Wallingford, UK [11]). Participants were required to place the oral fluid collector swab into a tube of buffer which contains sodium phosphates, salts, detergents and preservatives which act as extractant agents drawing the target analytes into the buffer. Researchers then obtained saliva samples from athletes and analyzed their saliva by adding the sample (saliva/buffer mixture) to a sample pad on a lateral flow device cassette. Salivary samples were ‘incubated’ for 10 min. Once the mixture was on the cassette it flowed across the membrane of the sample pad and the gold labelled anti-cortisol was captured by the cortisol test line, resulting in the appearance of a red line. The cassette is then placed into the lateral flow cube reader device which measured the lines intensity and converted this into a corresponding cortisol concentration within the saliva sample, providing an immediate result [11].

### 2.3. Measures of Stress, Optimism and Decision Making Skills

Players were also required to complete paper-based self-report questionnaires on their stress levels. These included Cohen’s Perceived Stress Scale [12]) which measures the perception of stress identifying the degree to which situations in one’s life are appraised as stressful [12]. Questions are totaled out of a maximum of 40, with scores above 20 indicating increased levels of stress. Levels of optimism (LOT-R [13]) assesses individual differences in generalized optimism versus pessimism [13]. Scores 15 and above show an increased tendency toward optimism. Decision-making skills were assessed using Raven’s Progressive Matrices [14]) which specifically measures non-verbal abstract and cognitive functioning [14]. Scores above 50 indicate above average levels of abstract reasoning. These measures were correlated to cortisol to determine if these specific psychological traits were correlated to participant cortisol levels.

### 2.4. Data and Statistical Analyses

Data was screened for normality prior to statistical analyses. Values less than 0.5 are indicative of poor reliability, values between 0.5 and 0.75 indicate moderate reliability, values between 0.75 and 0.9 indicate good reliability, and values greater than 0.90 indicate excellent reliability [15]. One-way ANOVA with Bonferroni post-hoc analyses were utilized to compare cortisol responses between low, medium and high responders. Cohen’s *d* effect sizes [16] were utilized to compare the magnitude of difference (small ≤ 0.2; moderate 0.21–0.8; large ≥ 0.81) between the three groups. Pearson correlation analyses were used to determine the relationships between self-reported psychological traits and baseline cortisol levels. Means (±SD) are presented, and alpha set at *p* < 0.05.

## 3. Results

### 3.1. Reliability of Cortisol Measures

All participants completed the cortisol measures without incident. Intraclass correlation coefficient analysis showed a good reliability between test-retest average measures (ICC = 0.793) [17]. Table 1 shows individual and group mean test-retest scores. All participants fell within the normative range for resting cortisol levels [18].

### 3.2. Cortisol Measures

**Figure 1:**
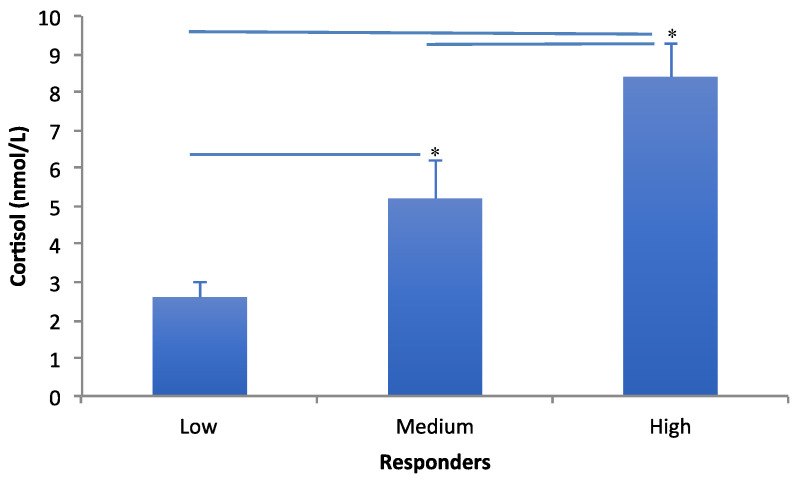
Mean cortisol (±SD) levels for ‘low’, ‘medium’ and ‘high’ responders. Asterisks (*) and lines indicate post-hoc significant difference between groups (*p* < 0.05).

Cortisol data between low, medium and high responders are illustrated in Figure 1. Group comparison revealed a significant difference between the three levels of responders (*F*_2,19_ = 69.00, *p* < 0.001) with post hoc testing showing the significant differences between all three groups (*p* < 0.001). Cohen’s *d* comparisons showed large effect size differences between low to medium (*d* = 3.4), and low to high (*d* = 8.3) responders, and medium to high (*d* = 3.4) responders.

### 3.3. Psychological Responses and Correlation to Cortisol

Table 2 illustrates individual psychological and group mean scores for optimism, stress and decision making. Correlations between players mean individual cortisol results and psychological traits are shown in Table 3. Correlations between variables were deemed low and no significant correlations were observed between cortisol levels and psychological traits measured.

## 4. Discussion

To the authors’ knowledge, this is the first report presenting tiered levels of baseline cortisol levels within a sample of professional athletes. Supporting our hypothesis, we classified three tiers identifying ‘low’, ‘moderate’, and ‘high’ responders. However, our secondary hypothesis that individual responses would correlate to psychological traits (optimism, stress levels, or decision making) was not supported. 

The sensitivity of cortisol and its responses to daily events such as medication, exercise, nutrition and stress are well described [19]. Thus, our objective in classifying responders, and therefore methodology, focused on basal cortisol testing, whereby athletes were instructed to take their measure within 15 min upon waking. Athletes in our test-retest analysis showed good reliability analysis, giving us confidence in addressing the question if athletes’ responders would fall into one of three tiers for basal cortisol response.

Our data shows that the all athletes fell within the overall resting levels for cortisol [20]. However, anecdotal evidence has also suggested that individuals will fall into ‘low’, ‘moderate’, and ‘high’ responders. Yet this classification has not been reported formally. Our data in a small but homogeneous cohort illustrates that individuals do fall into one of three categories of resting cortisol. We are, however, unable to provide a mechanistic reason for this tiered response. Given our good intra-individual reliability in cortisol measures, we can only suggest that this tiered response is genetic in nature [19].

The second hypothesis, that there would be a correlation between cortisol levels and psychological traits, was not supported. A reason for the lack of correlation found between cortisol levels and specific personality traits may be due to the fact that cortisol is a biological hormone, and variation in individual’s levels is likely due to a genetic predisposition [21], independent of psychology. Therefore, identifying specific psychological mechanisms which contribute to an individual’s cortisol expression may be difficult. While cortisol is affected by psychological factors such as stress and mood disorders, [22] the research to date on psychological traits with cortisol levels is equivocal [23]. Unlike previous studies in the general population, our study aimed to investigate a specific cohort who have similar physical training programs and experience similar psychosocial stress [24]. While we did not find a significant association between cortisol and personality traits, it is still important to stress the novel aspect of this study being an examination of an elite group of athletes who experience not only physical stressors of training, but also tolerating ongoing psychological stress on a near daily basis [25]. 

Whilst our study was limited to a convenience, and relatively small, sample it is important to note that our question focused on quantifying and classifying different responders within a relatively homogeneous cohort. Further studies should continue to investigate our initial findings, to further tease out this phenomenon of tiered responders. Future research should also incorporate time measures during the day to explore if tiered responses are still observed within the diurnal variations seen over the course of a day [18,26]. Finally, whilst the method of cortisol sampling was done using saliva samples, and therefore a quantitative measure, psychological traits were collected using self-report measures which, while psychometrically sound, may have potential to be manipulated by professional athletes who may not want to reveal their stress for fear of missing team selection, or other interpretations by coaching staff that may jeopardize their standing in the club [5]. This emphasizes the importance of implementing rigorous baseline testing of individuals’ salivary cortisol, to clearly quantify if an athlete is a low, moderate or high responder. Moreover, as there are links between cortisol and stress [22], knowing an athlete’s tier of response can provide an objective indication if an athlete is suffering from any psychological stress, particularly if their psychological reports do not show change.

The results of this study demonstrate that individual athletes will likely fall into either a low, average or high band of baseline cortisol. Importantly, while cortisol is a reliable biological measure of stress, it is essential that support staff within professional teams be mindful of overgeneralizing results of individuals athletes’ cortisol levels to their psychological profile. For example, an athlete being in the high band of cortisol does not mean that they have higher levels of stress than athletes in the low band. Therefore, while cortisol may be used as a measure to determine stress levels and help athletes understand their psychophysiological stress in a more objective way, overemphasizing cortisol levels could lead to misinterpretation about an individual and their current status. It is important cortisol results are rigorously ‘baselined’ to determine an athlete’s resting level, but also used in conjunction with other methods of analysis to form a holistic overview of a professional athlete’s status.

## Figures and Tables

**Table 1:** Correlations between cortisol levels to psychological traits.

Participant	Test 1 (nmol/L)	Test 2 (nmol/L)	Mean (nmol/L)
1	8.9	8.4	8.7
2	4.9	7.2	6.1
3	8.9	6.6	7.8
4	3.5	2.2	2.9
5	2.7	6.0	4.4
6	10.0	7.4	8.7
7	6.7	7.3	7.0
8	2.9	5.5	4.2
9	10.2	7.3	8.8
10	3.0	2.8	2.9
11	4.7	3.0	3.9
12	1.9	7.6	4.8
13	9.8	9.9	9.9
14	5.8	3.9	4.9
15	6.2	6.7	6.5
16	7.8	7.0	7.4
17	8.0	10.0	9.0
18	7.6	4.0	5.8
19	7.8	9.6	8.7
20	2.2	1.8	2.0
21	2.7	2.5	2.6
22	3.5	4.4	3.9
**Mean (±SD)**	**5.9 (±2.4)**	**6.0 (±2.4)**	**5.9(±2.4)**

**Table 2:** Individual total and group mean (±SD) psychological scores.

	Optimism (0–24)	Stress (0–40)	Decision Making (0–60)
1	9	10	51
2	9	13	48
3	11	14	35
4	11	15	48
5	15	19	50
6	11	17	45
7	11	19	43
8	10	23	43
9	9	22	28
10	9	23	35
11	12	18	53
12	14	21	53
13	8	19	45
14	10	18	31
15	10	15	49
16	13	19	47
17	15	16	47
18	8	20	41
19	12	19	49
20	13	16	45
21	11	22	37
22	10	14	50
**Mean (±SD)**	**11.0 (±2.0)**	**17.8 (±3.4)**	**44.2(±7.0)**

**Table 3:** Correlations between cortisol levels to psychological traits.

	Optimism	Stress	Decision Making
Pearson’s *r* ^1^	0.236	0.051	0.195
*p* Value	0.291	0.821	0.385

^1^ Bivariate calculation between cortisol and dependent variable.
